# Inclusion and use of ethical analysis in health technology assessment reports relevant to genomic newborn screening

**DOI:** 10.1017/S0266462326104115

**Published:** 2026-08-06

**Authors:** Siun Gallagher, Joanne Scarfe, Ainsley J. Newson, Sarah Norris

**Affiliations:** 1 Faculty of Medicine and Health, Sydney School of Public Health, Sydney Health Ethics, The University of Sydney, Australia; 2 Faculty of Medicine and Health, Sydney School of Public Health, The Leeder Centre for Health Policy, Economics and Data, The University of Sydney, Australia

**Keywords:** ethics, health technology assessment, genomics, genetics, newborn screening

## Abstract

**Objectives:**

Advances in the use of genomic technologies in newborn bloodspot screening (NBS) raise ethical challenges that should be systematically and critically considered. We examine how ethical analyses have been incorporated into health technology assessments (HTA) relevant to genomic NBS to inform how ethics might be incorporated in Australian HTAs of genomic NBS.

**Methods:**

We conducted an inductive content analysis of publicly available HTA reports and summaries relevant to genomic NBS published between January 2013 and June 2023 across 18 countries. Eligible reports related to genetic or genomic testing or population screening. These reports were screened for inclusion of ethical considerations and coded with NVivo for their presence, structure, and reported influence on decision making. Twelve reports with substantial ethical discussion were examined in-depth through qualitative thematic analysis.

**Results:**

One hundred and ninety reports met our inclusion criteria, with Spain, Australia, and the United Kingdom heavily represented. Ethics was mentioned in 39 percent of reports, and ethical analysis appeared to influence recommendations in 13 percent. When present, ethical considerations focused on equity, harm, and autonomy. Consideration of ethical issues was mostly unstructured, descriptive, and lacked normative conclusions.

**Conclusions:**

Although a range of ethical considerations were raised in the HTA reports relevant to genomic NBS, ethical analyses were generally inconsistently applied or reported, and the way in which ethics has influenced HTA recommendations was not always clear. Further work is needed to understand how the role of ethics in Australian HTAs of genomic NBS could be strengthened to ensure ethically defensible decisions.

## Background

Ethical considerations are inherent to health technology assessment (HTA), yet there is continuing debate about how ethics should be addressed and how ethical analyses are applied in HTA practices (1–4). Jurisdictions recognize the need to include ethics in HTA and subsequent decision making about new medical technologies (5), but the extent to which this occurs varies between HTA bodies and is influenced by expertise, committee culture, and context (6–8). Additionally, while contemporary HTA guidelines generally provide advice on the importance of including ethics, there is no consensus on how this should be done (1;9;10), and compliance with guidelines can be difficult in practice (11;12).

Genomics is one field where technology assessment frequently encounters ethical questions. (13–15). Additionally, standard methods for valuing nongenomic tests, such as assessing clinical- and cost-effectiveness, may be insufficient to capture these broader implications of genomic applications, or these concepts may require more rigorous interpretation and implementation (13).

These challenges will need to be addressed when decision makers come to assessing the use of genomic sequencing in newborn bloodspot screening (NBS). NBS is a long-established and highly trusted population screening program in high-income countries, designed to detect serious childhood-onset conditions within days of birth, enabling timely intervention to improve health outcomes for the child (16–19). Genomic sequencing technologies are advancing rapidly, and multiple research studies of genomic NBS (gNBS) are underway or complete (20;21). The inclusion of genomics in NBS could substantially expand its scope and allow detection of hundreds of additional genetic conditions, potentially improving outcomes for more children and families (20). Implementation of whole-genome sequencing into formal NBS programs has occurred in one Italian state (17;22–24) and is planned for the United Kingdom (25).

However, the use of genomics in NBS raises significant ethical, legal, and equity implications beyond those accepted for traditional NBS (26;27). These include the implications of the newborn’s genetic information for parents or relatives, risks to privacy, potential for discrimination, and psychological harms and inappropriate healthcare arising from uncertain or nonpenetrant findings (28;29).

Ethical considerations arising from the introduction of genomics into NBS must be appropriately addressed in HTA (17;20;26). Although there is a substantial literature on the challenge of undertaking ethical analysis in HTA, and on ethical considerations in gNBS, relatively few genomic HTA reports exist, and little has been written about how assessors and decision makers use (or should use) ethical analyses in assessments of gNBS (3;13;30). Similarly, although the relevant Australian HTA guidelines (10) detail the different approaches that might be relevant for undertaking an ethical analysis in the context of an HTA, there is little guidance on how such analyses might then be included in deliberations by the Medical Services Advisory Committee (MSAC) and decision making by government. This study sought to examine *whether* and *how* ethics has been addressed in HTAs related to genetic and genomic population screening and clinical testing, with the aim of identifying existing international practice in comparable jurisdictions that might be relevant for future Australian HTAs of gNBS. To this end, we focused on HTAs in areas closely adjacent to gNBS, genetic and genomic diagnostic testing and population screening, because we expected them to share relevant characteristics.

## Methods

We were interested in both procedural and methodological elements of relevant HTA reports and wanted to understand the extent of the representation of ethics and its influence on decision making. As there is no standardized approach for identifying or categorizing ethics content in HTA reports, we adopted an exploratory strategy guided by our research questions (3): (1) How is ethical analysis structured and considered in HTA reports relevant to genetic and genomic testing and population screening?; (2) What frameworks and theories are used to frame the ethical analysis in these reports?; (3) How do the reports express the results of the ethical analysis?; and (4) To what extent do the ethical analyses inform decision making? We also wished to explore whether and how the framing and presentation of an ethical analysis influences the uptake and use of the analysis.

### Search strategy

We reviewed publicly available national or multicountry HTA summaries published in English, and full reports for which an English language summary was available, published between January 2013 and June 2023. Full HTAs and rapid reviews were included. Reports were sourced from the websites of HTA agencies in countries that contribute to the International Network of Agencies for Health Technology Assessment (INAHTA), that have mature, publicly funded, or state-governed health systems and established HTA processes, and that publish at least summary reports in English. We included the UK National Screening Committee as the only agency in the United Kingdom with a role in assessing population and NBS proposals. Regional or subnational HTA reports were excluded, except for Spain, where nationally coordinated documentation from regional agencies is available. To ensure completeness, we emailed each HTA agency directly to verify our list and request any additional relevant reports. Additional details of the search strategy are provided in Supplementary Material.

### Inclusion and relevance screening

All reports were manually screened using the following terms: *gene*, *genetic*, *genomic*, *mutation*, *screen/screening*, *sequencing*, *test*, and *variant.* Reports that met the search criteria were downloaded. Full reports or summaries written in English were selected. Where English summaries were embedded in non-English reports, the full report was downloaded. Reports were then hand-screened for relevance by SG. See Supplementary Material for how we managed the variety of languages in our sample.

Eligible reports involved initiatives in the following categories: population screening; newborn screening; reproductive carrier screening; prenatal or preimplantation testing; diagnostic or predictive genetic/genomic testing; pharmacogenetics or pharmacogenomics; and genomic prognostic testing. Categories are described in Box 1.

Box 1. Properties of the categories of the reports included in the analysis.

- **Population screening:** screening of a population or population subgroup that shares one or more characteristics but is not defined by medical condition (e.g., pregnant women and men over 65 years) for disease or risk factors known to affect that population or population subgroup.
- **Prognostic testing:** assessments of a particular genetic sequencing technology (or group of like technologies for genomic sequencing) and gene testing as a general approach for a disease or group of diseases, for example, gene expression tests. This does not include gene/drug pairings.
- **Reproductive genetic carrier screening:** testing of prospective parents carried out before pregnancy or in early pregnancy for genetic variants known to cause serious heritable recessive or X-linked conditions.
- **Prenatal or preimplantation testing:** screening or targeted testing by any modality of embryos or fetuses to establish the chance that the future child will have a genetic condition.
- **Newborn screening:** population screening applied to newborns with a view to early detection of disorders by any testing modality, including genetic testing.
- **Pharmacogenetics/pharmacogenomics:** genetic testing for predicting response and/or tolerance to a (non-cancer) medication.
- **Diagnostic and predictive testing:** use of genetic or genomic testing with the primary aim of establishing a diagnosis. This includes diagnosis of childhood syndromes, molecular diagnosis of genetic conditions, diagnosis of conditions appearing in adulthood, and genetic risk assessment.

Reports were excluded if they focused on disease monitoring (e.g., screening for complications in people with diabetes), gene therapies, or standard test-treatment/companion technology pairings. For analysis, the most comprehensive English version of each relevant report was selected, imported into NVivo 14, and treated as a case. Each case was coded and assigned attributes according to key characteristics (Table 1).

**Table 1.** Key characteristics assigned to cases in NVivo Table 1. long description.

	Characteristic^a^	Description
1	Country	Country or jurisdiction of the HTA agency (or equivalent entity) responsible for the HTA report (or its equivalent).
2	Category	Intervention/program^b^ category (as defined in Box 1).
3	Type of report (integrated or stand-alone)	Whether ethics (or ethical issues) were included in the main body of the publicly available HTA report (integrated) or were the subject of a stand-alone report.
4	Language	Describes the version of report used in the analysis and its relationship to any available reports in the original language (in non-anglophone jurisdictions).
5	Jurisdictional HTA guidelines	The available HTA guidelines for each country were assessed for the level of comprehensiveness of their guidance on ethical analysis. The report was assigned an attribute that reflected the level that applied to its country of origin.
6	Organization of ethics	How ethics was presented in the report, for example, as a chapter or section; also gives an indication of the extent to which an ethics analysis was a focus for the report.
7	Ethics personnel on team	Whether or not this was specified, and an indication of the confidence level for this determination^c^.
8	Ethics – effect on decision^d^	Extent of the impact of ethics on the decision. This was assessed by reading relevant sections of the report.

a Characteristics 1–4 are descriptive; characteristic 2 cross references texts not included in the dataset; the attributes of characteristics 6–8 were assigned based on reading of the reports.

b Hereafter “intervention.”

c For example, short (one or two pages) summaries do not contain information on the HTA team.

d This feature of the report was included as a key characteristic to facilitate cross-tabulation and monitoring of the data during the process of analysis.

### Screening for ethics-related language

All relevant records were screened using NVivo’s text search query for the terms: ethic*; equit* (includes inequit*); moral*; harm* (excluding evaluations of clinical benefit and harm); principl*; justice; autonomy; and value* (excluding values associated with clinical or laboratory study variables and monetary value). We selected the screening terms based on our experience in conducting HTAs, familiarity with HTA reports and ethics literature, and preexamination of four reports with large ethics sections to ensure we captured how the terms were used in context. The reports were screened in their entirety for relevant ethics terms. Records containing any of the terms were selected for detailed coding for ethics themes. The remaining records were retained in the dataset and included in the quantitative analyses as “cases screening negative for ethics”.

### Analysis

We used inductive content analysis (ICA) due to its suitability for under-researched healthcare topics, its ability to generate rich, practice-oriented insights (31), and its support for mixed methods, especially where issue framing affects interpretation (32). ICA involves iterative coding, with the development of “content categories” (denoted as “themes” in the Results section) that are derived from, rather than imposed on, the data. We were interested primarily in the role ethics played in deliberation (ethics-informed decision making), but it was also necessary to examine how the reports described the methodological aspects of the evaluative procedure (ethical analysis). The “content categories” were derived from the data via a process of iterative coding, which was undertaken by SG using NVivo14, with AJN, SN, and JS participating in category development. SG read every reference to a “content category” to make sure that the ethics terms it included were being deployed in an ethical context. Ethics “content categories” were mapped to report sections to show whether concepts occurred in descriptive or analytic contexts. The same procedure was employed for the in-depth qualitative analysis of the selected reports.

### Selection of reports for ethics in-depth qualitative analysis

Based on our preliminary analysis, which showed ethical themes to be sparsely represented in our data, we undertook an in-depth qualitative analysis of the records in which the presence of ethics “content categories” was well represented. Based on a review of the coding patterns of the most highly coded records, we set a cut-off of 20 mentions of ethics “content categories” for identifying records (the “selected reports”) for inclusion in this analysis. A further nine reports had between ten and nineteen mentions of ethics and related concepts; however, this ethics-related material was too limited to provide additional insights on the conduct and impact of ethical analysis.

## Results

We present our findings in two parts: a description of the characteristics of the included reports, and answers to the focused research questions.

### Characteristics of included reports

One hundred and ninety reports from eighteen countries met our inclusion criteria: ninety-two full reports in English and ninety-eight reports with English-language executive summaries (Table 2). The category with the most reports (seventy-four) was *population screening.* There were eighteen records concerning *newborn screening* (Table 2).

**Table 2.** Number of reports in each category, by country, 2013–2025 Table 2. long description.

Country	Population screening^a^	Diagnostic or predictive testing	Prognostic testing	Prenatal or preimplantation testing	Newborn screening	Pharmaco-genetics or pharmaco-genomics	Reproductive genetic carrier screening	Total number of reports
Spain	36	8	5	4	4	1	0	**58**
Australia	0	18	3	4	2	0	2	**29**
UK	15	1	1	3	5	1	0	**26**
South Korea	1	11	0	1	0	3	0	**16**
Germany	9	0	0	1	4	0	0	**14**
Canada	3	5	2	0	0	1	1	**12**
France	2	1	2	0	1	0	0	**6**
Wales	0	2	2	0	0	2	0	**6**
Norway	1	0	1	2	0	0	0	**4**
Malaysia	4	0	0	0	0	0	0	**4**
Belgium	0	0	2	1	0	0	0	**3**
Ireland	1	0	1	0	1	0	0	**3**
Austria	1	1	1	0	0	0	0	**3**
Sweden	0	0	0	2	0	0	0	**2**
Italy	0	0	1	0	0	0	0	**1**
Regional or global	0	0	0	1	0	0	0	**1**
Thailand	1	0	0	0	0	0	0	**1**
Finland	0	0	0	0	1	0	0	**1**
Total	**74**	**47**	**21**	**19**	**18**	**8**	**3**	**190**

a Excluding newborn screening and reproductive genetic carrier screening.

### Categories where ethics is mentioned

The report categories with most mentions of ethics were *reproductive carrier screening*, *newborn screening*, and *prenatal or preimplantation testing.* Significant numbers of reports in the latter two categories did *not* address ethics (Table 3).

**Table 3.** Representation of ethics in the different categories of HTA reports Table 3. long description.

Categories	Ethics mentioned		No ethics mentioned		Total
	Number of reports	% of the reports in this category	Number of reports	% of the reports in this category	
Reproductive genetic carrier screening	3	100%	0	0%	3
Newborn screening	11	61%	7	39%	18
Prenatal or preimplantation testing	11	58%	8	42%	19
Prognostic testing	9	43%	12	57%	21
Diagnostic or predictive testing	20	43%	27	57%	47
Population screening^a^	19	26%	55	74%	74
Pharmacogenetics & pharmacogenomics	2	25%	6	75%	8
**Total**	**75**	39%	**115**	61%	**190**

a Excluding newborn screening and reproductive genetic carrier screening.

### HTA guidelines

Almost all (169/190) of the reports were undertaken in jurisdictions where current HTA guidelines include guidance regarding ethics. Guidelines were not publicly available for sixteen reports.

### Reports that mentioned ethics and organization of ethics

Fewer than half the reports (75/190) mentioned ethics or ethics-related themes (hereafter “ethics”). The themes observed were equity, benefits, harms, weighing benefit and harm, autonomy and choice, reproductive decision making, confidentiality, vulnerability, public opinion and acceptability, consumer impact, epistemic concerns, respect for persons, blame and stigma, values, accountability, and feasibility (Supplementary Table 1).

Three of the reports were stand-alone ethical analyses, two had separate ethics chapters, thirteen included ethics sections, eight had paragraphs with “ethics” in the title, and forty-nine mentioned ethics under other headings (Supplementary Table 2). Twelve records were selected for in-depth qualitative analysis (Supplementary Table 3).

### Answers to the focused research questions

#### How is ethical analysis structured and considered in HTA reports?

In 65 percent (49/75) of “ethics positive” reports, ethical issues appeared without a dedicated section (Supplementary Table 2). While short English-language summaries of original reports (which may include more comprehensive ethics coverage) limit inferences that can be drawn, fifteen of the ninety-eight non-English reports (15 percent) mentioned ethics compared with sixty of ninety-two (66 percent) full English-language reports.

Of the seventy-five reports referencing ethics, fifty-four addressed these issues in analysis (e.g., findings or discussion) sections (Table 4). Ethics also appeared in terms of reference, scope, or methods (including ethics as part of a literature review), and the conclusion (if provided). Some reports referred to ethics in additional sections, such as reports of consultations, interviews, or submissions, and accounts of the deliberations of subcommittees or the recommending committee.

**Table 4.** Representation of ethics in different sections of HTA reports Table 4. long description.

	Location of references to ethics	Number of reports
Ethics in project design	Project scope includes ethics	30
	Method contains ethics steps	33
	Ethics literature review undertaken	16
	Ethical theory specified	11
Ethics in description, analysis and discussion	Executive summary refers to ethics	11
	Ethical issues in findings and discussion	54
	Conclusion takes ethics into account	22

In some instances, ethical considerations were reproduced in the executive summary (Table 4), although they were sometimes excluded even if they featured prominently elsewhere in the report. A few English-language summaries of non-English-language reports introduced ethical considerations not present in the main text.

Some reports contained a clearly identifiable ethical analysis section – generally incorporated in a “Results” section, along with the PICO (population, intervention, comparator, and outcome) and economic analyses. Such ethics sections typically appeared late in reports, after the PICO and economic analysis, and were generally shorter in length. In several reports, ethics was situated alongside sections addressing patient, social, or operational issues, with frequent cross-referencing. For example, discussions on consent often appeared in both ethical and operational contexts.

Processes that involved subcommittees as well as technical writing teams tended to consider (sometimes different) ethical issues in separate report sections. In some reports, notably those from Australia, it was possible to see ethics discussed in several ways in different contexts. Twenty-two reports (eighteen Australian) described stakeholder submissions or consultations (e.g., from patient groups or professional bodies), which frequently mentioned ethical considerations. However, these considerations were not always reflected in later stages of the assessment or incorporated in an ethics section or summaries of committee deliberations. The Australian reports contained a section describing the recommending committee’s deliberations on the findings from other sections of the report.

There was variability in the use of academic ethics or bioethics literature: some analyses consisted solely of the results of a literature review, others cited relevant literature to support key points, while others did not draw on any ethics literature.

#### What frameworks and theories are used to frame the ethical analysis?

Eleven reports explicitly identified an ethical theory (Supplementary Table 4). Of these, five applied Beauchamp and Childress’ four principles approach (33) and six referenced public health ethics approaches (34–36). In a few cases, the chosen theory structured the presentation of the analysis. Additionally, seven reports used HTA-specific procedural frameworks or checklists, such as Hofmann’s Socratic approach (9) instead of or alongside ethical theory. Beyond these, several reports reflected on the process of ethical reasoning itself. Five described their work as “applied ethics analysis,” while another five acknowledged the methodological challenges of conducting ethical analysis. Some also explained the purpose of ethical reasoning and the procedure the ethical analysis would adopt.

#### How are the results of the ethical analysis expressed?

In reports with ethics literature reviews and in some other reports with substantial ethics sections, discussion of ethics tended to be confined to these sections, and the analysis was presented in the “voice” of the technical writing team. Other HTAs occasionally reported in a voice based on the discussions about ethics held by the recommending committee and/or subcommittees. In some cases, the use of multiple voices affected the report’s clarity.

Where ethics formed a major part of a report, a comprehensive list of relevant ethical considerations tended to be provided and discussed. Only a minority of reports ranked or compared the ethical issues identified or proposed mitigations for them. Even where multiple steps preceded the recommending body’s deliberations, ethical analysis was sometimes presented without clear prioritization of the ethical issues, or assessment relative to clinical or economic evidence.

#### To what extent do ethical analyses inform decision making?

Overall, there was limited information on how ethics influenced recommendations. Ethical considerations were given as part of the decision rationale in twenty-four reports (13 percent). Except the category *reproductive genetic carrier screening*, only a minority of records in each category reported a clear influence of ethics on the decision (Table 5).

**Table 5.** Influence of ethical considerations on the recommendation, by category Table 5. long description.

Category	Newborn screening	Population screening	Prognostic testing	Diagnostic and predictive testing	Pharmacogenetics and pharmacogenomics	Prenatal or preimplantation testing	Reproductive genetic carrier screening	Total
Ethical considerations influence recommendation	6	7	0	5	1	3	2	24
Total number of records in the category	**74**	**47**	**21**	**19**	**18**	**8**	**3**	**190**

Report recommendations were clearly set out to decision makers, but were often non-committal regarding ethics. In five reports, the recommendation took the form: “a range of ethical factors need to be considered if the decision maker opts to approve this application.” One stated that an ethical perspective could not inform the application’s approval. This suggests that there is not always a requirement (or willingness) to answer the question: “Is introducing this intervention/technology ethically justified?.” Some reports, notably those following the pattern in Supplementary Material Box S1, showed greater decisiveness in making value judgements and critically appraised individual ethical concerns. Some reports included constructive suggestions for addressing complex issues and referenced existing policies and duties to support decision making. If it was featured in a recommendation, ethics was generally limited to one specific issue, even if several issues were discussed elsewhere in the report.

We examined whether there were correlations between evidence that ethical considerations had influenced the decision or recommendation and (i) the availability of comprehensive ethics guidance in the HTA guidelines of the relevant jurisdiction; (ii) the inclusion of statements about ethics in the methods section of the report; (iii) the amount of ethics content; and (iv) the presence of dedicated ethics expertise on the team. We found no correlation with respect to the first two factors, and except ethical analysis presented in a stand-alone report or a full ethics chapter, there was no association between the amount of ethics content and reporting of ethics-informed decisions. Our ability to conclude the impact of dedicated ethics expertise was limited by the relatively small number of reports (37) listing team members and roles.

The inclusion or exclusion of ethics in final report recommendations did not appear to be influenced by the nature of the issue under consideration or by the position taken on the proposal for funding.

## Discussion

This study examined whether and how ethical analyses were integrated within a selection of “gNBS adjacent” HTA reports to inform how ethics might be integrated into future Australian HTAs for gNBS. We sought to understand the extent of representation of ethical themes, their reported influence on decision making, and how the framing and presentation of ethical analyses influenced their uptake and use in decision making. Although most relevant to HTA for gNBS, our findings have broader implications for how ethical questions are addressed in HTAs of genetic and genomic testing, and population screening.

References to ethics were absent in the majority of reports, consistent with broader observations about the relative absence of ethics in HTA more generally (38). When ethics was present, the reports included limited insights regarding how ethical issues were prioritized in deliberations: ethics appears to be neither elevated above other topics, nor downgraded, with ethical factors neither gateways nor barriers for decision making.

Importantly, the limited reporting of ethical assessments and deliberations by HTA committees in our set of reports does not mean that such activities did not take place, and indeed, committees can spend considerable time on ethics and other nonquantifiable factors (39;40). Nonetheless, this under-reporting makes it difficult for those on the outside to determine whether these processes were adequate or absent.

### Approaches to ethical considerations

We identified issues regarding the quality of ethical analysis in many reports, and a general reluctance by HTA report authors to engage with the implications of ethical analysis. Integrating ethical analysis in HTAs is recognized as important (38;41) and will be particularly relevant in gNBS given ongoing debates in this field (41;42). While a recent study suggests that ethics sections in full HTA reports are underpinned by literature (which should not be confused with ethical analysis) (3), dissatisfaction with the attention ethics receives in HTAs has been widely expressed (6;11;12;30;40–44). Earlier research identified the need for improved models for ethical analyses (including making ethics more visible and better integrated) and better incorporation of ethics expertise (6;12;41). There are also concerns that analyses are descriptive rather than critical (46), and that ethics is mentioned only in passing (6;46;47). The absence of agreed models for ethical analysis (1;41;46) and the unsettled roles of ethical theory and ethics expertise (41) are also relevant here.

Normative or evaluative findings were infrequent in our sampled reports. Ethical concerns were generally not ranked or weighed, nor was there clarification as to how identified ethical issues related to other dimensions. The tendency for stakeholder submissions or consultations, which often raise ethical considerations, not to be included in later stages of assessment or in ethics sections and committee deliberations raises important questions about the epistemic status of ethical testimony provided by patients and community stakeholders. While some mitigation strategies for ethical concerns were noted, most reports took a neutral stance that left decision makers with little substantive guidance. Ideally, ethical analysis in HTA should address whether a technology is ethically justified. While not all ethical analyses in HTA will result in normative conclusions, in our view, assessments should aim to present the decision maker with either a normative conclusion or a clear evaluation of the issues.

### Implications for Australian HTA practice

Our findings underscore the importance of transparent and complete reporting of ethical analysis and deliberation in HTAs of genetic and genomic tests, to better enable tracking and evaluation of ethical reasoning through the assessment process. This is consistent with observations by others for HTAs more generally, where ethics can be seen as a specialist concern, and where a clearly assigned responsibility for ethical analysis is often absent (3;16;46). However, absence of evidence of deliberation may provide a misleading picture of what counts as ethically salient, especially as reports may reflect the institutional values of the drafting team and HTA agencies (48) rather than the recommending committee (49).

HTA treats facts and values separately (49;50) and we recognize that a full ethical analysis may not be necessary for every HTA (37). Given the instrumental role that HTA plays in public funding decisions, HTA methods are necessarily grounded in quantifiable or testable evidence (40;42;47), but more is needed to support the transparent inclusion of ethics in HTA, especially in areas of health (e.g., NBS), and for types of technologies (e.g., genomics) with clear ethical questions.

Reluctance to engage with the implications of ethical analyses may reflect under-specification in HTA procedures. While our local HTA guidelines for investigative technologies (10) provide guidance on how to do an ethical analysis, there is currently no model or guideline in Australia for how such analyses should be translated and applied in our deliberative decision-making processes. This is important, because the procedures and governance structure employed by HTA bodies influence both the inclusion and organization of ethics in a report. Any future model, template or guideline would need to account for procedural (e.g., reaching agreement on which assessment require an ethical analysis; clarifying the roles of all participants in the process) and substantive (e.g., organization of ethical issues within a report; handling of consultation feedback) aspects, as well as our multiple phases of HTA (i.e., PICO/protocol development by PASC, assessment by ESC, deliberation by MSAC, and documentation by the Department of Health, Disability and Ageing). Consideration should also be given to including a qualified ethicist as a participant in the assessment of ethically complex cases.

Enabling robust incorporation of ethical considerations into HTA for gNBS should also attend to existing normative and methodological constraints, including ethicists as “professional strangers” in HTA, assumptions that economic analysis covers off ethical issues, and the amorphous or even intangible nature of bioethics methodology (38). However, as Hofmann argues, health is inherently ethical, and even if it is hard to integrate ethics, this does not mean it should not occur (38).

### Limitations

This study was limited by the public availability of HTA records. For practical reasons, we included only reports or summaries in English. In cases where English summaries were brief, we screened samples of the original language reports using Google Translate and found strong alignment in how ethics was represented. Variability in agency scope and the exclusion of regional reports from federal systems (unless part of a coordinated national process) also contributed to sample irregularities. Extending our search to include reports from the UK National Screening Committee also contributed to sample irregularity (emphasizing anglophone countries) but was justified by the unavailability, otherwise, of relevant records from this high-volume producer of HTA reports. We acknowledge that despite our best efforts to identify reports written in languages other than English (including contacting every agency to identify missing reports), we may have missed reports that meet our inclusion criteria.

A substantial proportion of the dataset (98/190 reports) consisted of English-language executive summaries rather than full reports. As ethical considerations may be abbreviated or omitted in summaries, this may have led to an underestimation of the prevalence and depth of ethical analysis. This may represent a potentially important source of bias. The dataset was also weighted toward a small number of jurisdictions, particularly Spain, Australia, and the United Kingdom. This reflects the availability of publicly accessible HTA reports but may also capture differences in national HTA practices, reporting conventions, and the visibility of ethical analysis in reports, rather than necessarily representing broader international trends.

Our focus on publicly available reports does not allow our research to establish with certainty the contributions of commitment to ethical analysis, its execution, or its reporting to the deficiencies we identified. However, our findings’ similarity to published literature on HTA broadly suggests a multifactorial etiology. Qualitative studies focusing on decision-making bodies and their participants would be helpful in advancing knowledge in this area.

## Conclusion

Although this study was focused on HTAs relevant to gNBS, our findings highlight multiple opportunities for countries like Australia to improve both how they incorporate ethical reasoning into decision making in HTAs of medical technologies, and how it reports on this. These implications highlight the need for us to develop and implement HTA guidelines or tools that prioritize ethical concerns, support ethical analysis, and ensure its integration into deliberation, recommendations, and decision making in ways that are commensurate with the complexity of an ethical issue. With gNBS on the horizon, there is an urgent need to ensure ethical issues and value judgements are more explicit, and that ethical reasoning is integrated into HTAs in a more structured and transparent way.

## Supporting information

10.1017/S0266462326104115.sm001 Gallagher et al. supplementary material Gallagher et al. supplementary material

## Data Availability

A list of the reports selected for in-depth qualitative analysis is available in Supplementary Table 3. A file containing all 190 reports included in the analysis is available on request.

## Supplementary material

The supplementary material for this article can be found at http://doi.org/10.1017/S0266462326104115.

## Long descriptions

### Table 1. Long description

The table consists of three columns: an unlabelled index column, Characteristic, and Description.

* Row 1: Country. Description: Country or jurisdiction of the H T A agency or equivalent entity responsible for the H T A report or its equivalent.

* Row 2: Category. Description: Intervention or program category as defined in Box 1.

* Row 3: Type of report (integrated or stand-alone). Description: Whether ethics or ethical issues were included in the main body of the publicly available H T A report (integrated) or were the subject of a stand-alone report.

* Row 4: Language. Description: Describes the version of report used in the analysis and its relationship to any available reports in the original language in non-anglophone jurisdictions.

* Row 5: Jurisdictional H T A guidelines. Description: The available H T A guidelines for each country were assessed for the level of comprehensiveness of their guidance on ethical analysis. The report was assigned an attribute that reflected the level that applied to its country of origin.

* Row 6: Organization of ethics. Description: How ethics was presented in the report, for example, as a chapter or section; also gives an indication of the extent to which an ethics analysis was a focus for the report.

* Row 7: Ethics personnel on team. Description: Whether or not this was specified, and an indication of the confidence level for this determination.

* Row 8: Ethics – effect on decision. Description: Extent of the impact of ethics on the decision. This was assessed by reading relevant sections of the report.

Navigate back to Table 1..

### Table 2. Long description

The table contains 19 rows of data including a final total row. The columns are: Country, Population screening, Diagnostic or predictive testing, Prognostic testing, Prenatal or preimplantation testing, Newborn screening, Pharmaco-genetics or pharmaco-genomics, Reproductive genetic carrier screening, and Total number of reports.

* Spain: 36 population, 8 diagnostic, 5 prognostic, 4 prenatal, 4 newborn, 1 pharmaco, 0 carrier. Total: 58.

* Australia: 0 population, 18 diagnostic, 3 prognostic, 4 prenatal, 2 newborn, 0 pharmaco, 2 carrier. Total: 29.

* U K: 15 population, 1 diagnostic, 1 prognostic, 3 prenatal, 5 newborn, 1 pharmaco, 0 carrier. Total: 26.

* South Korea: 1 population, 11 diagnostic, 0 prognostic, 1 prenatal, 0 newborn, 3 pharmaco, 0 carrier. Total: 16.

* Germany: 9 population, 0 diagnostic, 0 prognostic, 1 prenatal, 4 newborn, 0 pharmaco, 0 carrier. Total: 14.

* Canada: 3 population, 5 diagnostic, 2 prognostic, 0 prenatal, 0 newborn, 1 pharmaco, 1 carrier. Total: 12.

* France: 2 population, 1 diagnostic, 2 prognostic, 0 prenatal, 1 newborn, 0 pharmaco, 0 carrier. Total: 6.

* Wales: 0 population, 2 diagnostic, 2 prognostic, 0 prenatal, 0 newborn, 2 pharmaco, 0 carrier. Total: 6.

* Norway: 1 population, 0 diagnostic, 1 prognostic, 2 prenatal, 0 newborn, 0 pharmaco, 0 carrier. Total: 4.

* Malaysia: 4 population, 0 diagnostic, 0 prognostic, 0 prenatal, 0 newborn, 0 pharmaco, 0 carrier. Total: 4.

* Belgium: 0 population, 0 diagnostic, 2 prognostic, 1 prenatal, 0 newborn, 0 pharmaco, 0 carrier. Total: 3.

* Ireland: 1 population, 0 diagnostic, 1 prognostic, 0 prenatal, 1 newborn, 0 pharmaco, 0 carrier. Total: 3.

* Austria: 1 population, 1 diagnostic, 1 prognostic, 0 prenatal, 0 newborn, 0 pharmaco, 0 carrier. Total: 3.

* Sweden: 0 population, 0 diagnostic, 0 prognostic, 2 prenatal, 0 newborn, 0 pharmaco, 0 carrier. Total: 2.

* Italy: 0 population, 0 diagnostic, 1 prognostic, 0 prenatal, 0 newborn, 0 pharmaco, 0 carrier. Total: 1.

* Regional or global: 0 population, 0 diagnostic, 0 prognostic, 1 prenatal, 0 newborn, 0 pharmaco, 0 carrier. Total: 1.

* Thailand: 1 population, 0 diagnostic, 0 prognostic, 0 prenatal, 0 newborn, 0 pharmaco, 0 carrier. Total: 1.

* Finland: 0 population, 0 diagnostic, 0 prognostic, 0 prenatal, 1 newborn, 0 pharmaco, 0 carrier. Total: 1.

* Totals: 74 population, 47 diagnostic, 21 prognostic, 19 prenatal, 18 newborn, 8 pharmaco, 3 carrier. Grand Total: 190.

Navigate back to Table 2..

### Table 3. Long description

The table consists of six columns: Categories, Ethics mentioned (Number of reports and percentage), No ethics mentioned (Number of reports and percentage), and Total.

* Reproductive genetic carrier screening: 3 reports (100 percent) mentioned ethics; 0 reports (0 percent) did not. Total: 3.

* Newborn screening: 11 reports (61 percent) mentioned ethics; 7 reports (39 percent) did not. Total: 18.

* Prenatal or preimplantation testing: 11 reports (58 percent) mentioned ethics; 8 reports (42 percent) did not. Total: 19.

* Prognostic testing: 9 reports (43 percent) mentioned ethics; 12 reports (57 percent) did not. Total: 21.

* Diagnostic or predictive testing: 20 reports (43 percent) mentioned ethics; 27 reports (57 percent) did not. Total: 47.

* Population screening (excluding newborn and reproductive carrier screening): 19 reports (26 percent) mentioned ethics; 55 reports (74 percent) did not. Total: 74.

* Pharmacogenetics and pharmacogenomics: 2 reports (25 percent) mentioned ethics; 6 reports (75 percent) did not. Total: 8.

* Total across all categories: 75 reports (39 percent) mentioned ethics; 115 reports (61 percent) did not. Grand total: 190.

Navigate back to Table 3..

### Table 4. Long description

The table consists of three columns: Category, Location of references to ethics, and Number of reports.

Under the category Ethics in project design:

- Project scope includes ethics: 30 reports.

- Method contains ethics steps: 33 reports.

- Ethics literature review undertaken: 16 reports.

- Ethical theory specified: 11 reports.

Under the category Ethics in description, analysis and discussion:

- Executive summary refers to ethics: 11 reports.

- Ethical issues in findings and discussion: 54 reports.

- Conclusion takes ethics into account: 22 reports.

Navigate back to Table 4..

### Table 5. Long description

The table consists of nine columns and two data rows. The columns represent different genetic testing categories and a total.

* Newborn screening: 6 records influenced out of 74 total.

* Population screening: 7 records influenced out of 47 total.

* Prognostic testing: 0 records influenced out of 21 total.

* Diagnostic and predictive testing: 5 records influenced out of 19 total.

* Pharmacogenetics and pharmacogenomics: 1 record influenced out of 18 total.

* Prenatal or preimplantation testing: 3 records influenced out of 8 total.

* Reproductive genetic carrier screening: 2 records influenced out of 3 total.

* Total: 24 records influenced out of 190 total.

Navigate back to Table 5..
