# Supplementary material for: Inclusion and use of ethical analysis in health technology assessment reports relevant to genomic newborn screening
Source: Int J Technol Assess Health Care. 2026 Aug 6;42(1):e84. doi: 10.1017/S0266462326104115 (PMC13735823; doi:10.1017/S0266462326104115)
Supplement: Supplementary file 1 [file S0266462326104115sup001.docx]

**INCLUSION AND USE OF ETHICAL ANALYSIS IN HEALTH TECHNOLOGY ASSESSMENT REPORTS RELEVANT TO GENOMIC NEWBORN SCREENING**

Siun Gallagher^1^, Joanne Scarfe^2^, Ainsley J. Newson^1^, Sarah Norris^2^

*1. The University of Sydney, Faculty of Medicine and Health, Sydney School of Public Health, Sydney Health Ethics, Sydney, New South Wales, Australia*

*2. The University of Sydney, Faculty of Medicine and Health, Sydney School of Public Health, The Leeder Centre for Health Policy, Economics and Data, Sydney, New South Wales, Australia*

**Supplementary Materials**

**METHODS**

*How we managed the variety of languages in our sample*

In addition to English language reports and stand-alone executive summaries of reports prepared in other languages, our search yielded reports entirely in their original language, and English language executive summaries that were embedded in or easily linked with accessible full original language reports. This represented a dilemma, as although our intention was to include the most comprehensive English language report available, we suspected that the executive summaries, because of their brevity, would be less likely to cover ethics considerations than full reports. Wishing to test this, we used translations of key terms in our codes (provided by native Spanish and German speakers and by SG, who is an intermediate French speaker) to search the original language reports. We found that there was a high correlation between the representation of ethics terms in the summaries and the full original language versions – chiefly because of the low representation of ethics terms. On the basis of this test, we concluded that the benefit of working with the full reports in their original language was insufficient to justify the cost and labour involved in obtaining and working with translations.

**RESULTS**

**Supplementary Table 1. Description and frequency of ethics-related themes**

| Themes* | Description | Examples of the types of issues that define the theme | Number of records containing this theme | Number of instances of this theme |
| --- | --- | --- | --- | --- |
| Equity | A concern for the effect of the proposal on equity. | A decision to approve funding for an intervention previously available privately would correct inequity; operational barriers to providing an intervention to all who need it would generate inequity. | 36 | 122 |
| Harms | Concerns that the intervention would bring about harms that are unrelated to side effects or known clinical consequences of the intervention. Psychological harms dominated this subtheme. | Numerous reports refer to psychological harms associated with undergoing testing and awaiting results, as well as with receiving bad news, and several refer unsolicited findings as a harm. | 33 | 92 |
| Autonomy and choice | A concern about the potential of the initiative to limit or disregard individuals’ autonomy, and with ensuring that implementation fosters the individual’s capacity to make autonomous choices. Autonomous decisions, informed consent, and the provision of information and counselling were frequently considered together. | A report on non-invasive prenatal testing (NIPT) for trisomies 21, 18 and 13 offers a typical formulation, highlighting the ethical issues a person considering having the test would need to aware of and proposing adequate pre- and post-test counselling to ‘ensure’ an informed decision on whether to proceed. | 30 | 105 |
| Weighing benefit and harm | Concerns an ethical aim of ensuring that benefits outweigh harms, or an evaluation of the overall benefit-harm ratio of the proposed intervention (excluding harm-benefit evaluations focused solely on the clinical effects of the intervention). | A report on non-invasive prenatal testing (NIPT) for trisomies 21, 18 and 13 states that it is hard to weigh up the benefits in terms of the reproductive decisions the test enables with the psychological harms related to the test or ensuing decisions. | 17 | 45 |
| Reproductive decision-making | A principal value of genetic and genomic testing in the context of reproduction, enabling decisions or choices that are informed by knowledge about whether one or both members of the couple possess a pathogenic variant, and offering the possibility of wider familial reproductive benefits arising from this information. | A report on reproductive carrier testing for cystic fibrosis, spinal muscular atrophy and fragile X syndrome identified as principal benefits both immediate reproductive outcomes and favourable effects on reproductive choice. | 16 | 62 |
| Benefits | Concerns beneficence as an aim and the achievement of extra-clinical benefits, such as the end of the diagnostic odyssey. | A report assessing screening familial hypercholesterolaemia in childhood claimed that the case for approving the intervention is supported by the secondary benefits accruing to a wider group as a result of cascade testing in families of children who screen positive. | 14 | 23 |
| Feasibility | A concern with the availability of the technology and skilled staff needed to ensure the intervention can be rolled out safely and equitably. | A report on the ethical dimensions of genome-wide sequencing claimed that for practice in this area to be ethical, equitable and harm-free, those involved must have the right skills, knowledge, and commitments. | 14 | 19 |
| Consumer impact, public opinion and acceptability | A desire to act in accordance with empirical data (where available) on consumer and public acceptability of the intervention. | A report on expanded reproductive carrier testing considered that the committee was not satisfied with the evidence offered on ethical and societal acceptability. | 13 | 17 |
| Confidentiality | A concern for the preservation of data in ways that maintain confidentiality, and also for ensuring that individuals undergoing testing are adequately informed about the ramifications of decisions in relation to release of their data to others to whom it might be relevant, for example family members. | Ethical dilemmas may arise when physicians must balance complying with the wishes of patients who wish their carrier or affected status to be kept confidential and their own duty of care to prevent disease, which would involve informing family members of their risk of having a child with a hereditary disease. | 13 | 24 |
| Epistemic concerns | Concerns about the quality of the available evidence on the benefits and harms of the intervention. | Concern was expressed in reports on the digitisation of breast screening about the status of the evidence for its accuracy, safety, and effectiveness. | 12 | 13 |
| Blame and stigma | Concerns about the parental blame and guilt for passing on pathogenic variants and fear about the family and affected individuals being stigmatised. | Societal fears that individuals may be defined by their genetic status and that such deterministic perceptions will affect life choices of those with pathogenic variants. | 11 | 20 |
| Respect for persons | Concerns that testing for heritable conditions and their consequences breach the principle of respect for persons through failing to treat them as whole persons regardless of their abilities and/or considering them only in terms of their economic costs. | Some reports refer to the complex challenge of valuing diverse individual lives while also suggesting that they confer burdensome costs on society. | 9 | 11 |
| Value or values | Desire to act in accordance with empirical data (where available) on patient, family, and societal values and the values that guide policy. | A report on DNA Mismatch Repair Deficiency Tumour Testing for Patients with Colorectal Cancer included a section concerning patients’, families’ and caregivers’ values in relation to the test. | 6 | 9 |
| Accountability | Acknowledgement of the accountability of physicians for their decisions. | A report concerning the use of AI in mammographic test interpretation highlighted how algorithms might disrupt accountability. | 2 | 2 |
| Vulnerability | Concern about the vulnerability of newborns, who rely on parental decision-making, and the vulnerability of parents at that time. | A report concerning the addition of SCID to the newborn screening program took into consideration the possibility that parents’ post-birth emotional vulnerability might impact on their ability to provide consent. | 2 | 4 |
| Total |  |  | 64 | 568 |

^*^ The ethical aspects were handled as categories in the ICA but are referred to here as ‘themes’ to avoid confusion with the categories of HTA reports cited in Box 2.

**Supplementary Table 2. Organisation of ethics within the report**

| Organisation of ethics | Number of reports mentioning ethics | % of the total number of reports mentioning ethics | Number of references | Number of references per report |
| --- | --- | --- | --- | --- |
| No section, but incidental mention of ethics | 49 | 65% | 200 | 4 |
| Paragraph | 8 | 11% | 87 | 11 |
| Section with ethics heading | 13 | 17% | 212 | 16 |
| Chapter | 2 | 3% | 63 | 31 |
| Standalone document | 3 | 4% | 114 | 38 |
|  | **Total: 75** | **100%** | **Total: 676** | **Average: 9** |

**Supplementary Table 3. Reports selected for in-depth qualitative analysis (‘selected reports’)**

| Jurisdiction | HTA Agency | Title | Categories | Year |
| --- | --- | --- | --- | --- |
| Australia | MSAC | *Newborn bloodspot screening for X-linked adrenoleukodystrophy* | Newborn Screening | 2023 |
| Australia | MSAC | *Expanded reproductive carrier testing of couples for joint carrier status of genes associated with autosomal recessive and X-linked conditions* | Reproductive genetic carrier screening | 2022 |
| Australia | MSAC | *Genetic testing for childhood hearing impairment* | Diagnostic and predictive | 2022 |
| Australia | Medical Services Advisory Committee (MSAC) | *Genetic testing for heritable kidney disease (other than Alport syndrome)* | Diagnostic and predictive | 2021 |
| Australia | MSAC | *Reproductive carrier testing for cystic fibrosis, spinal muscular atrophy and fragile X syndrome* | Reproductive genetic carrier screening | 2020 |
| Austria (full report in English) | HTA Austria Austrian Institute for Health Technology Assessment GmbH | *Genetic Testing in the Context of Familial Hypercholesterolaemia Management: Organisational and ethical implications and economic aspects* | Diagnostic and predictive | 2020 |
| Canada | CADTH | *Genome-Wide Sequencing: Ethical Considerations* | Diagnostic and predictive | 2019 |
| Canada | Canadian Agency for Drugs and Technologies in Health (CADTH) | *HPV Testing for Primary Cervical Cancer Screening: A Health Technology Assessment* | Population screening | 2019 |
| EuNeTHA | European Network for Health Technology Assessment | *Screening of foetal trisomies 21, 18 and 13 by noninvasive prenatal testing* | Prenatal or preimplantation testing | 2018 |
| Ireland | Health Information and Quality Authority | *Health Technology Assessment of the addition of severe combined immunodeficiency (SCID) to the National Newborn Bloodspot Screening Programme* | Newborn screening | 2023 |
| Ireland | Health Information and Quality Authority | *Health technology assessment of human papillomavirus testing as the primary screening method for prevention of cervical cancer* | Population screening | 2017 |
| UK | National Screening Committee | Child-family screening for familial hypercholesterolemia: Ethical issues | Population screening | 2022 |

i. A further 9 reports had between 10 and 19 mentions of ethics and related concepts but did not contain sufficient ethics-related material to enable insights to be drawn on the conduct and impact of the ethical analysis.

**Supplementary Table 4. Ethical theories, guidelines, and checklists referenced in the reports**

| **Theory, guideline, or checklist** | **Instances** | **Jurisdiction** | **HTA Agency** | **Title** | **Year** |
| --- | --- | --- | --- | --- | --- |
| **Theoretical approaches** | | | | | |
| **Beauchamp and Childress’s four principles approach**[1] | 5 | Australia | MSAC | *Reproductive carrier testing for cystic fibrosis, spinal muscular atrophy and fragile X syndrome* | 2020 |
|  |  | Canada | Canadian Agency for Drugs and Technologies in Health (CADTH) | *HPV Testing for Primary Cervical Cancer Screening: A Health Technology Assessment* | 2019 |
|  |  | Canada | CADTH | *Genome-Wide Sequencing: Ethical Considerations* | 2019 |
|  |  | EuNeTHA | European Network for Health Technology Assessment | *Screening of foetal trisomies 21, 18 and 13 by noninvasive prenatal testing* | 2018 |
|  |  | Ireland | Health Information and Quality Authority | *Health Technology Assessment of the addition of severe combined immunodeficiency (SCID) to the National Newborn Bloodspot Screening Programme* | 2023 |
| **Public health ethics approaches** | 6 | Australia | MSAC | *Newborn bloodspot screening for X-linked adrenoleukodystrophy* | 2023 |
|  |  | Canada | Canadian Agency for Drugs and Technologies in Health (CADTH) | *HPV Testing for Primary Cervical Cancer Screening: A Health Technology Assessment* | 2019 |
|  |  | Canada | CADTH | *Genome-Wide Sequencing: Ethical Considerations* | 2019 |
|  |  | Ireland | Health Information and Quality Authority | *Health technology assessment of human papillomavirus testing as the primary screening method for prevention of cervical cancer* | 2017 |
|  |  | Malaysia | Health Technology Assessment Section (MaHTAS), Ministry of Health Malaysia | *HPV DNA-based screening test for cervical cancer* |  |
|  |  | UK | National Screening Committee | Child-family screening for familial hypercholesterolemia: Ethical issues | 2022 |
| **Frameworks and checklists** | | | | | |
| **Hofmann’s Socratic approach** |  | Austria | HTA Austria Austrian Institute for Health Technology Assessment GmbH | *Genetic Testing in the Context of Familial Hypercholesterolaemia Management: Organisational and ethical implications and economic aspects* | 2020 |
| **Hofmann’s Socratic approach** [2] **combined with Burls et al.’s framework for tackling ethical issues in HTA** [3] | 2 | Canada | Canadian Agency for Drugs and Technologies in Health (CADTH) | *HPV Testing for Primary Cervical Cancer Screening: A Health Technology Assessment* | 2019 |
| **HTA Core Model® for Rapid Relative Effectiveness Assessment (EUnetHTA, n.d.) Core Model** | 2 | EuNeTHA | European Network for Health Technology Assessment | *Screening of foetal trisomies 21, 18 and 13 by noninvasive prenatal testing* | 2018 |
|  |  | Ireland | Health Information and Quality Authority | *Health Technology Assessment of the addition of severe combined immunodeficiency (SCID) to the National Newborn Bloodspot Screening Programme* | 2023 |
| **Kass’s Ethical framework process** [4] | 1 | Ireland | Health Information and Quality Authority | *Health technology assessment of human papillomavirus testing as the primary screening method for prevention of cervical cancer* | 2017 |
| **SBU assessment method** [5] | 2 | Sweden | Swedish Agency for Health Technology Assessment and Assessment of Social Services  (SBU) | Prenatal Diagnosis through Chromosomal Microarray Analysis (CMA) | 2016 |
|  |  | Sweden | SBU | *Prenatal Diagnosis through Next Generation Sequencing (NGS)* | 2016 |

**Box S1 Characteristics of the ethics analysis in reports in which ethics appeared to influence decision-making**

**Personnel**

- Ethicist on team

**Method**

- Relevant ethical framework named, clearly defined and used throughout
- Evaluates against standard (clearly articulated principles)
- Engages in a consultation expressly on ethical issues
- Seeks and clearly takes into account patient, family, and stakeholder views (and shows how they’re taken into account)
- Analysis is shaped by the chosen ethical framework

**Discussion**

- Ethical argumentation (not mere listing of possible ethical issues) aiming towards a result
- Proposes solutions to the identified problems (so that the initiative can be ethically implemented)
- Clearly weighs population and individual benefits
- Presents a concrete and comprehensive discussion of potential ethical problems
- Cites appropriate reports, policies, and authoritative literature
- Draws on ethical concepts in order to help identify the right course of action
- Thinks through synergies and consequences for other programs
- Considers personal, family, community, and population benefits/harms

**Conclusion**

- Makes normative statements (though still presents issues for consideration by the decider)
- Comes to a definitive position in cases where there might be a number of different views
- Calls on the duties of deciders
- Identifies further work that needs to be done in order to inform ethical decisions
- Thinks through synergies or side effects for other programs

**REFERENCES**

[1]	Beauchamp TL, Childress JF. *Principles of Biomedical Ethics*. Oxford: Oxford University Press, 1979.

[2]	Hofmann B, Droste S, Oortwijn W, et al. Harmonization of ethics in health technology assessment: a revision of the Socratic approach. *Int J Technol Assess Health Care* 2014; 30: 3–9.

[3]	Burls A, Caron L, Langavant GC de, et al. Tackling ethical issues in health technology assessment: A proposed framework. *International Journal of Technology Assessment in Health Care* 2011; 27: 230–237.

[4]	Kass NE. An ethics framework for public health. *Am J Public Health* 2001; 91: 1776–1782.

[5]	Swedish Agency for Health Technology Assessment and Assessment of Social Services. (n.d.). *SBU Method*. Retrieved 24 February 2026, from https://www.sbu.se/en/method2/
